# Prevalence and Landscape of Pathogenic or Likely Pathogenic Germline Variants and Their Association With Somatic Phenotype in Unselected Chinese Patients With Gynecologic Cancers

**DOI:** 10.1001/jamanetworkopen.2023.26437

**Published:** 2023-07-31

**Authors:** Hao Wen, Qin Xu, Xiujie Sheng, Huawen Li, Xipeng Wang, Xiaohua Wu

**Affiliations:** 1 Department of Gynecologic Oncology, Fudan University Shanghai Cancer Center, Shanghai, China; 2 Department of Oncology, Shanghai Medical College, Fudan University, Shanghai, China; 3 Clinical Oncology School of Fujian Medical University, Fujian Cancer Hospital, Fuzhou, China; 4 Department of Gynecology, The Third Affiliated Hospital of Guangzhou Medical University, Guangzhou, China; 5 Department of Gynecology, Zhuhai People’s Hospital (Zhuhai Hospital Affiliated with Jinan University), Zhuhai, China; 6 Department of Obstetrics and Gynecology, Xinhua Hospital Affiliated to Shanghai JiaoTong University School of Medicine, Shanghai, China

## Abstract

**Importance:**

Understanding germline and somatic status in patients with gynecologic cancers could improve risk assessment and guide therapeutic decision-making.

**Objective:**

To evaluate the prevalence and landscape of germline pathogenic or likely pathogenic (P/LP) variants and explore whether these variants are associated with somatic phenotypes and cancer risk in unselected patients with gynecologic cancers.

**Design, Setting, and Participants:**

This cross-sectional study retrospectively enrolled unselected patients in China with a gynecologic cancer, including ovarian, cervical, and endometrial, who underwent tumor-normal sequencing using a 520-gene panel from October 1, 2017, through May 31, 2021.

**Exposure:**

Germline variants in gynecologic cancers.

**Main Outcomes and Measures:**

The P/LP germline variant rates in 62 cancer predisposition genes were assessed using descriptive statistics. The associations of P/LP variant status with age, somatic profiles, and cancer risk were also investigated using the Fisher exact test or Student *t* test.

**Results:**

A total of 1610 women (median [IQR] age, 54 [47-62] years; 1201 [74.6%] with stage III-IV disease) were included (945 with ovarian cancer, 307 with endometrial cancer, and 358 with cervical cancer). The prevalence of patients with P/LP variants was 20.5% (194 of 945) for ovarian cancer, 13.4% (41 of 307) for endometrial cancer, and 6.4% (23 of 358) for cervical cancer; 95.1% of the germline findings (n = 252) were potentially actionable, mainly in homologous recombination repair (HRR) and mismatch repair genes. Chinese patients with endometrial cancer had a higher rate of P/LP variants than a White population from The Cancer Genome Atlas (42 of 307 [13.7%] vs 24 of 367 [6.5%]; *P* = .003). In endometrial and cervical cancers, the prevalence of P/LP variants was 12.7% (30 of 237) and 4.8% (13 of 270), respectively, in patients diagnosed at age 45 years or older and increased to 25.0% (9 of 36; *P* = .09) and 12.0% (10 of 83; *P* = .04), respectively, for those with an onset age of less than 45 years. Mismatch repair P/LP variants were associated with a younger age at onset for ovarian cancer (46 vs 54 years; *P* = .02) and endometrial cancer (48 vs 57 years; *P* < .001), while HRR P/LP variants were associated with a younger age at onset for cervical cancer (46 vs 52 years; *P* = .04). Carriers of HRR P/LP variants had more prevalent somatic *TP53* variants and less common somatic variants in oncogenic driver genes vs noncarriers. *BRCA1/2* P/LP variants were also associated with moderate risks for endometrial and cervical cancer.

**Conclusions and Relevance:**

This study delineates the landscape of germline P/LP variants in Chinese women with gynecologic cancers. The findings highlight the hereditary factor in cervical cancer that has long been neglected and suggest the importance of next-generation sequencing–based genetic testing with a large gene panel for gynecologic cancers.

## Key Points

**Question:**

What is the prevalence and landscape of pathologic or likely pathologic (P/LP) germline variants, and are they associated with somatic phenotype in unselected patients with gynecologic cancers?

**Findings:**

This cross-sectional study of 1610 unselected patients with a gynecologic cancer revealed a P/LP germline variant prevalence of 20.5% for ovarian cancer, 13.4% for endometrial cancer, and 6.4% for cervical cancer, 95.1% of which were in homologous recombination repair and mismatch repair genes. These germline findings also revealed tumor lineage and gene-dependent associations with the age of disease onset and somatic phenotype.

**Meaning:**

These findings highlight the hereditary factor in cervical cancer, which has long been neglected and raises the importance of next-generation sequencing–based genetic testing with a large gene panel for gynecologic cancers.

## Teaser

This cross-sectional study describes the landscape of germline variants in gynecologic cancers and explores their association with somatic phenotypes and cancer risk.

## Introduction

Cancers of the female genital tract include ovarian, corpus uteri (mainly endometrial), cervical, vulvar, and vaginal tumors. These gynecologic tumors account for 15% to 20% of cancer-related deaths in women worldwide,^1,2,3,4,5^ with cervical, uterine, and ovarian cancers being the 3 most prevalent. The 2022 cancer statistics for China reported increasing incidence rates for cancers of the cervix and uterus and an increased mortality rate for cervical cancer during the past decade.^6^

Women harboring germline pathogenic/likely pathogenic (P/LP) variants have a higher risk of developing cancers, including gynecologic cancers, and at an earlier age of onset. Approximately 10% to 20% of ovarian cancers and 5% of endometrial cancers are inherited and attributable to deleterious germline variants.^7,8,9,10,11,12^ Autosomal dominant germline variants in genes, including *BRCA1* and *BRCA2* and those involved in the mismatch repair (MMR) pathway, are associated with an increased risk of developing hereditary breast and ovarian cancer syndrome and Lynch syndrome, respectively.^13^ The lifetime risk of developing ovarian cancer is estimated at 1.5% for the general population^14^ and significantly rises to 40% to 60% for *BRCA1* and 11% to 30% for *BRCA2* pathogenic variant carriers.^7^ Individuals harboring a deleterious germline variant in 1 of the MMR genes have a 20% to 70% lifetime risk of developing endometrial cancer.^15^ In comparison, cervical cancer is more sporadic and associated with genital infection with oncogenic types of human papillomavirus; however, the risk of developing minimal deviation adenocarcinoma, a rare variant of cervical cancer, increases in carriers of *STK11* germline variants who have Peutz-Jeghers syndrome.

The use of next-generation sequencing (NGS) has contributed to our current understanding of germline and somatic alterations involved in cancer development. Next-generation sequencing enables the simultaneous detection of clinically relevant variants and other genomic signatures that could guide therapy. With the improving turnaround time and cost-effectiveness of NGS, its application in clinical practice has grown in the past decade. However, the need for multigene genetic testing in gynecologic cancers remains limited due to the scarcity of clinically relevant genetic markers. The European Society of Molecular Oncology Precision Medicine Working Group released recommendations for the use of large NGS oncopanels for patients with metastatic cancers, including ovarian and endometrial cancer.^16^ Understanding the germline and somatic status of these patients could improve risk assessment and therapeutic management. Germline variants could shape the somatic landscape,^17,18^ which could also affect tumor biology, development, and progression.^19^

In this study, we investigated the landscape of germline P/LP variants in cancer predisposition genes in a large cohort of unselected Chinese women diagnosed with gynecologic cancers, including ovarian, endometrial, and cervical. To better understand the mechanism underlying the tumorigenesis of gynecologic cancers that develop in germline carriers, we also explored the association of germline variants with somatic profiles and cancer risk.

## Methods

### Patients

This cross-sectional study included 1610 Chinese women diagnosed with gynecologic cancers who submitted biological samples for genomic sequencing between October 1, 2017, and May 31, 2021. All eligible patients provided paired white blood cells for sequencing in parallel with tumor samples, including tumor biopsy, malignant pleural effusion, cerebrospinal fluid, or plasma. The study was approved by the ethics committee of the Fudan University Shanghai Cancer Center. All patients provided written informed consent to use their clinical information for research purposes. The study followed the Strengthening the Reporting of Observational Studies in Epidemiology (STROBE) reporting guideline.

### Next-Generation Sequencing

Paired white blood cells and tumor samples were sequenced using a 520-gene panel (Burning Rock Biotech), including 62 cancer predisposition genes (eTable 1 in Supplement 1). Details on NGS and the data analysis are provided in the eMethods in Supplement 1.

### Cancer Predisposition Analysis

The association between P/LP germline variants in certain genes and cancer was assessed using the public gnomAD database^20,21^ as the reference control, which contains exome sequencing data from 125 748 unrelated individuals. The race and ethnicity–matched East Asian subset was included in this analysis.

### Statistical Analysis

The data analysis was performed using R, version 4.1.0 software (R Foundation for Statistical Computing). Clinical characteristics were summarized as descriptive statistics. Fisher exact test and paired 2-tailed Student *t* test were used to compare groups. Odds ratios (ORs) and corresponding 95% CIs were estimated by Fisher exact test. Statistical significance was defined as a 2-sided *P* < .05.

## Results

### Patient Characteristics

Of the 1610 women included in the study, 945 (58.7%) were diagnosed with ovarian cancer, 358 (22.2%) with cervical cancer, and 307 (19.1%) with endometrial cancer (eFigure 1 in Supplement 1). Most women (1201 [74.6%]) had stage III to IV disease (Table 1). The cohort’s median age was 54 years (IQR, 47-62 years).

**Table 1.** Clinical Characteristics of the Cohort

Clinical feature	No. (%)				*P* value
	Overall (N = 1610)	Ovarian (n = 945)	Endometrial (n = 307)	Cervical (n = 358)	
Age, median (IQR), y	54 (47-62)	54.0 (47-62)	57.0 (51-64)	51 (45-58)	<.001
ND	151 (9.4)	112 (11.9)	34 (11.1)	5 (1.4)	
Clinical stage					
I	80 (5.0)	32 (3.4)	34 (11.1)	14 (3.9)	<.001
II	261 (16.2)	132 (14.0)	59 (19.2)	70 (19.6)	
III	699 (43.4)	458 (48.5)	115 (37.5)	126 (35.2)	
IV	502 (31.2)	268 (28.4)	92 (30.0)	142 (39.7)	
ND	68 (4.2)	55 (5.8)	7 (2.3)	6 (1.7)	
MSI status					
MSI-H	64 (4.0)	14 (1.5)	43 (14.0)	7 (2.0)	<.001
MSI-L	1 (0.1)	0	1 (0.3)	0	
MSS	1277 (79.3)	788 (83.4)	224 (73.0)	265 (74.0)	
ND	268 (16.6)	143 (15.1)	39 (12.7)	86 (24.0)	

Abbreviations: MSI-H, high microsatellite instability; MSI-L, low microsatellite instability; MSS, microsatellite stable; ND, no data.

### Germline P/LP Variants Across Gynecologic Cancers

**Figure 1.**
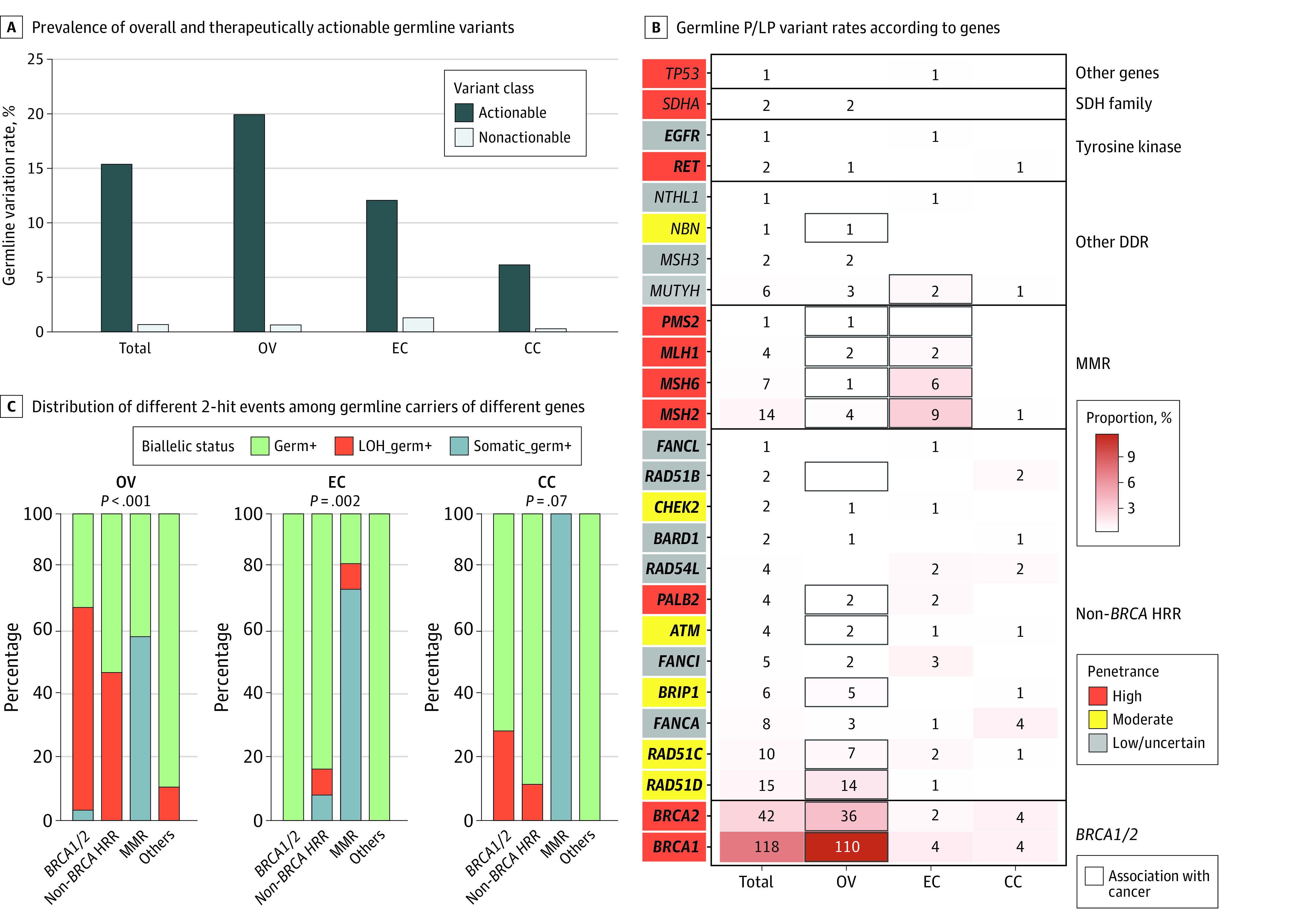
Prevalence of Germline Pathogenic/Likely Pathogenic (P/LP) Variants Across Gynecologic Cancers Therapeutic actionable genes are shown in boldface in panel B. The penetrance of genes was adopted from Srinivasan et al.^18^ CC indicates cervical cancer; DDR, DNA damage response; EC, endometrial cancer; HRR, homologous recombination repair; LOH, loss of heterozygosity; MMR, mismatch repair; OV, ovarian cancer; SDH, succinate dehydrogenase.

A total of 265 germline P/LP variants were detected in 26 cancer predisposition genes from 258 of the 1610 patients (16.0%) (Figure 1A; eTable 2 in Supplement 1). The prevalence was the highest in patients with ovarian cancer (20.5% [194 of 945]), followed by endometrial cancer (13.4% [41 of 307]) and cervical cancer (6.4% [23 of 358]). Among the 265 P/LP variants, most were frameshifts (129 [48.7%]) and occurred in *BRCA1* (118 [44.5%]) and *BRCA2* (42 [15.8%]) (eTables 3 and 4 in Supplement 1). Notably, 95.1% of these variants (n = 252) were potentially therapeutically actionable, with a prevalence of 15.3% (n = 247) (Figure 1A), most of which were located in homologous recombination repair (HRR) and MMR genes (Figure 1B). We found 77.0% of P/LP variants (n = 204) in genes associated with the cancer type where they were identified, and 23.0% (n = 61) had no canonical association with the cancer.

A total of 200 germline P/LP variants spanning 20 genes were detected in 194 patients with ovarian cancer (eTables 2 and 4 in Supplement 1). Six patients (3.1%) harbored 2 heterozygous variants in 2 genes. Most P/LP variants detected in ovarian cancer occurred in HRR genes (183 [91.5%]) (Figure 1B; eTable 3 in Supplement 1). Eight patients (4.1%) had P/LP variants located in MMR genes. Fifteen P/LP variants (7.5%) were identified in genes that lack canonical association with ovarian cancer.

A total of 42 germline P/LP variants spanning 18 genes were detected in 41 patients with endometrial cancer (eTables 2 and 4 in Supplement 1). One patient harbored 2 heterozygous variants in *EGFR* and *MSH2*. In addition to canonical Lynch syndrome–associated MMR genes (17 [40.5%]), P/LP variants in HRR genes, which are not canonically associated with endometrial cancer, also represented 47.6% of germline findings in endometrial cancer (n = 20) (Figure 1B; eTable 3 in Supplement 1).

We identified 23 germline P/LP variants spanning 14 genes from 23 patients with cervical cancer (eTables 2 and 4 in Supplement 1). The HRR variants comprised 86.9% of all germline findings (n = 20) in cervical cancer. *BRCA1*, *BRCA2*, and *FANCA* were the top 3 commonly altered genes, the P/LP variants of which were detected in 4 patients (17.4%) each (Figure 1B; eTable 3 in Supplement 1). The prevalence of P/LP variants was 8.5% (18 of 212) in squamous cervical cancer and 4.4% (3 of 68) in adenocarcinoma cervical cancer. The difference was not significant.

Next, we investigated the distribution of 2-hit events (also referred to as biallelic inactivation) in carriers of different P/LP variants (Figure 1C). In ovarian cancer, 69.6% (78 of 112) of *BRCA1/2* germline-variant tumors had 2-hit events, 94.9% (74 of 78) of which were via loss of heterozygosity (LOH). Similarly, 48.5% (16 of 33) of the ovarian cancers harboring non-*BRCA* HRR P/LP variants had 2-hit events solely via LOH. In contrast, 60.0% (3 of 5) of MMR germline-variant ovarian cancers presented with 2-hit events only by acquiring second somatic variants. The 2-hit rates in both HRR and MMR P/LP variants were significantly higher than that in the background of benign germline variants (HRR: 64.8% vs 20.3% [*P* < .001]; MMR: 60.0% vs 17.2% [*P* < .001]) (eFigure 2 in Supplement 1). In endometrial cancer, 2-hit events were seen in 83.3% (10 of 12) of MMR germline-variant tumors mainly through a second somatic variant (90.0% [9 of 10]) and only in 16.7% (2 of 12) of the tumors carrying non-*BRCA* HRR P/LP variants via either mechanism (Figure 1C). Notably, none of the 5 germline carriers of *BRCA1/2* P/LP variants had 2-hit events, suggesting that these variants might be incidental findings. The enrichment of 2-hit events in P/LP variants was only significant for MMR (83.3% [10 of 12] vs 13.1% [11 of 84]; *P* < .001) but not for HRR genes (11.8% [2 of 17] vs 17.7% [47 of 265], *P* > .99) (eFigure 2 in Supplement 1). In cervical cancer, we also identified LOH in 28.6% (2 of 7) of *BRCA1/2* and 11.1% (1 of 9) of non-*BRCA* HRR germline-variant tumors. The 2-hit rate for *BRCA* P/LP variants was numerically higher than the background (28.6% [2 of 7] vs 14.5% [43 of 297]; *P* = .33) (eFigure 2 in Supplement 1). The difference was not significant, which may be due to the small sample size. The 1 patient with cervical cancer harboring an MMR (*MSH2*) LP variant acquired a second somatic variant, conferring a 100% 2-hit rate compared with that of 9.9% in the background (*P* = .02).

### Comparison of the Rate of Germline P/LP Variants Between Different Populations

We further compared the prevalence of germline P/LP variants between this study’s cohort and the White population from The Cancer Genome Atlas (TCGA) research network.^22^ The overall rate of P/LP variants, based on the 53 overlapping genes between panels, was similar in ovarian cancer between cohorts, but the *BRCA2* P/LP variants were less frequent (36 of 945 [3.8%] vs 27 of 348 [7.8%]; *P* = .005) in our cohort than in TCGA (eFigure 3 in Supplement 1). Our endometrial cancer cohort had a significantly higher overall rate (42 of 307 [13.7%] vs 24 of 367 [6.5%]; *P* = .003) and more common P/LP variants in non-*BRCA* HRR genes (14 of 307 [4.6%] vs 6 of 367 [1.6%]; *P* = .04) than the TCGA data set. Patients with cervical cancer had comparable germline prevalence across genes between cohorts.

### Association Between Age and Status of Germline P/LP Variants

**Figure 2.**
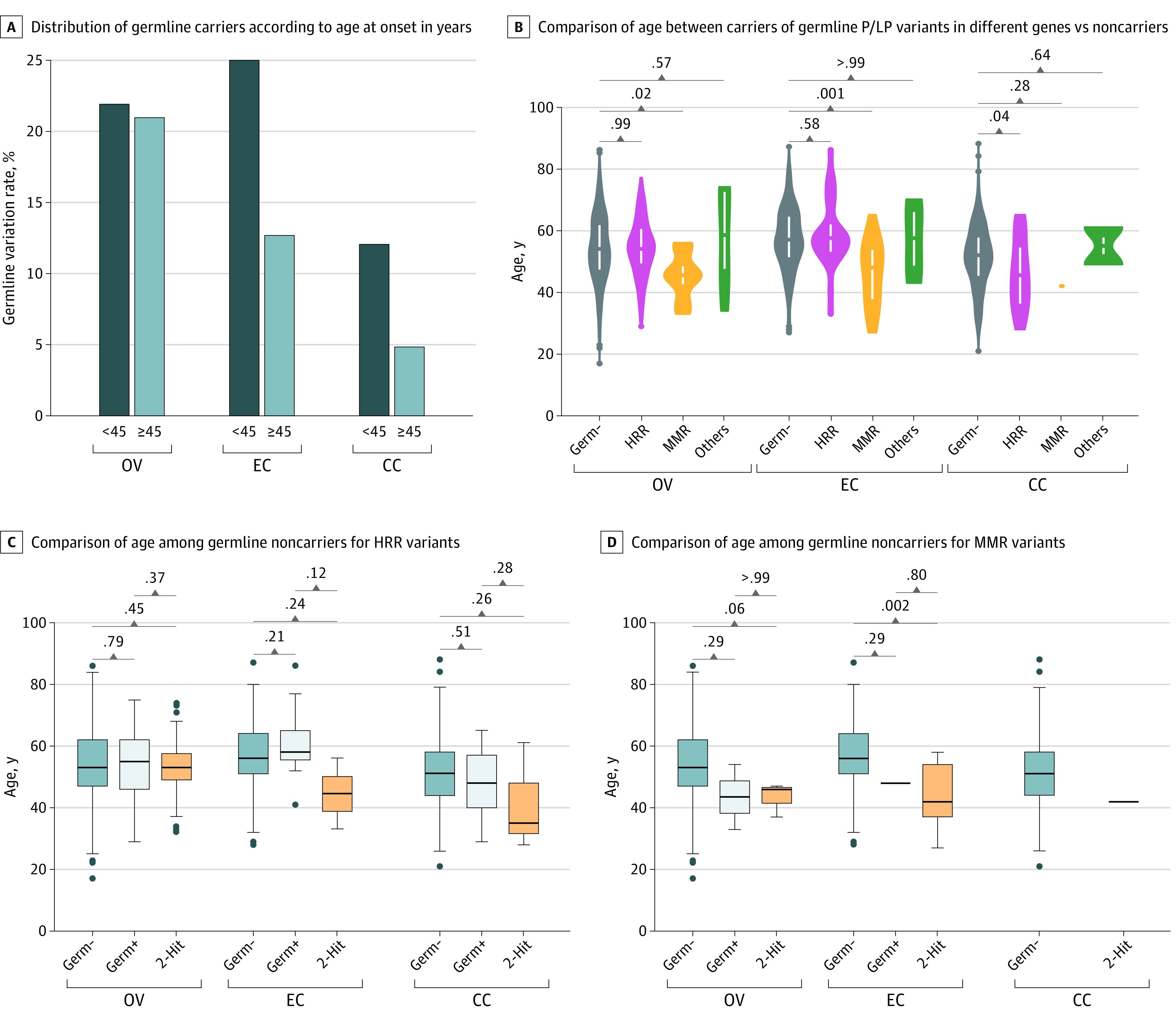
Association Between Age at Diagnosis and Germline Variant Status Across Gynecologic Cancer Types In panel B, vertical white lines represent 95% CIs, individual dots are abnormal values, and the width of the shape represents the frequency of the value. In panels C and D, the upper and lower bounds of the boxes are the upper and lower quartiles, whiskers represent 95% CIs, the center lines are the medians, and individual dots are abnormal values beyond the 95% CIs. In panel D, the single horizontal lines represent only a single value for those entries. CC indicates cervical cancer; EC, endometrial cancer; germ–, germline noncarriers; germ+, germline carriers without 2-hit events; HHR, homologous recombination repair; MMR, mismatch repair; OV, ovarian cancer; P/LP, pathogenic/likely pathogenic.

Patients with endometrial cancer and cervical cancer who were younger had a higher prevalence of P/LP variants. Specifically, the prevalence was 25.0% (9 of 36) in patients with early-onset (defined as age at diagnosis <45 years) endometrial cancer vs 12.7% (30 of 237) in those diagnosed at 45 years or older (*P* = .09). In cervical cancer, the prevalence was 12.0% (10 of 83) in patients with early onset and 4.8% (13 of 270) in those diagnosed at 45 years or older (*P* = .04) (Figure 2A). In contrast, the prevalence of P/LP variants was independent of age in ovarian cancer.

We stratified the cohort according to the status of P/LP variants in different gene sets. Compared with noncarriers, carriers of MMR P/LP variants were younger at onset of ovarian cancer (46 vs 54 years; *P* = .02) and endometrial cancer (48 vs 57 years; *P* < .001), while those with HRR P/LP variants were younger at onset of cervical cancer (46 vs 52 years; *P* = .04) (Figure 2B).

We further stratified the cohort according to the biallelic status of the P/LP variants. No significant difference was observed in age at onset among patients with different statuses for HRR variants in ovarian, endometrial, or cervical cancer (Figure 2C). Harboring MMR P/LP variants with 2-hit events was significantly associated with early onset in endometrial cancer, and a similar but nonsignificant result was seen in ovarian cancer (Figure 2D). The only patient with cervical cancer who harbored an MMR LP variant had a 2-hit event and was diagnosed at age 42 years. Of note, this analysis is limited by the small sample size, especially those with 2-hit events.

### Distinct Somatic Variant Profiles and Signatures in Carriers of Germline P/LP Variants

**Figure 3.**
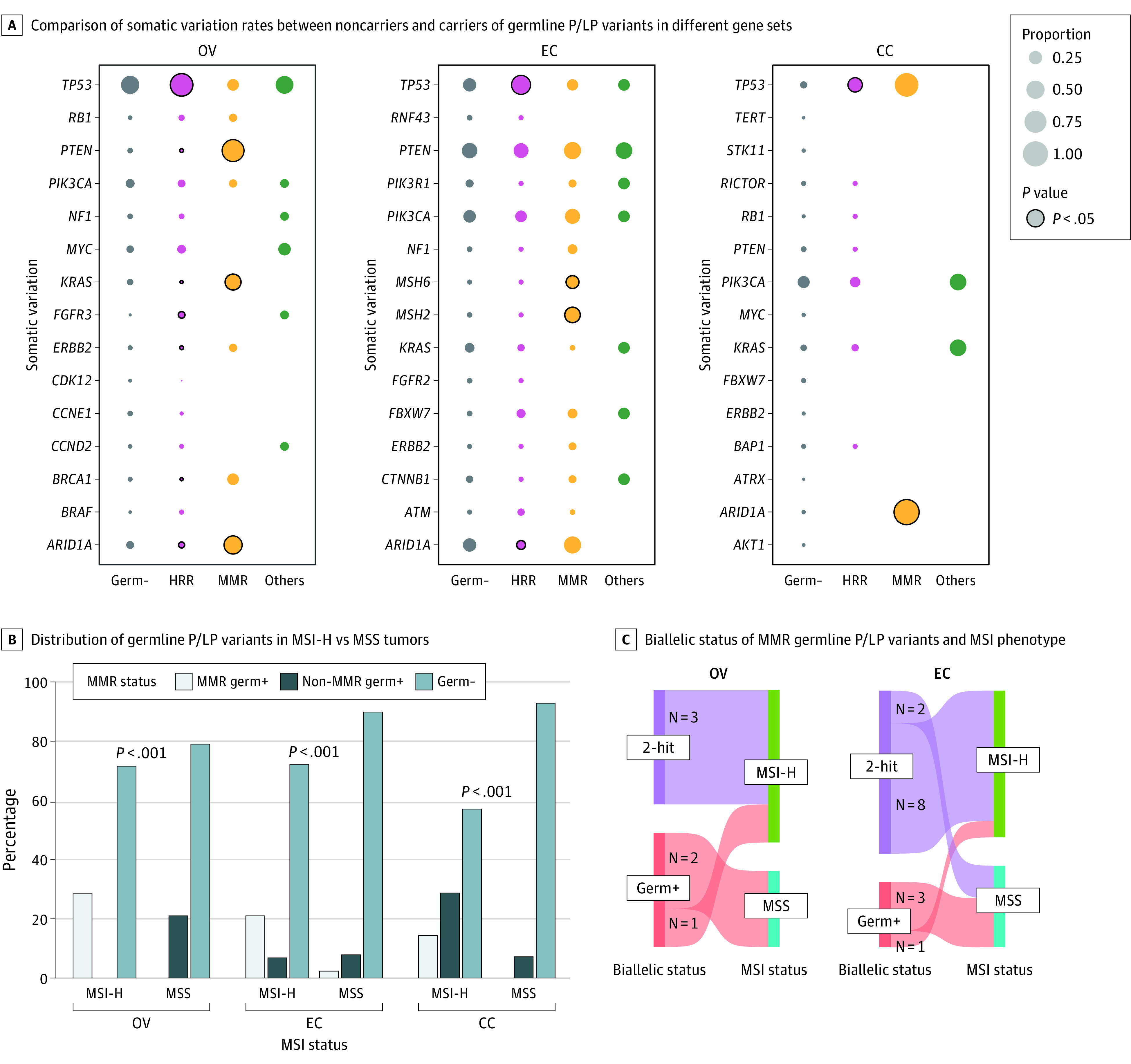
Association of Germline Pathogenic/Likely Pathogenic (P/LP) Variants With Somatic Profiles and Signatures CC indicates cervical cancer; EC, endometrial cancer; germ–, germline noncarriers; germ+, germline carriers without 2-hit events; HRR, homologous recombination repair; MMR, mismatch repair; MSI, microsatellite instable; MSI-H, high microsatellite instability; MSS, microsatellite stable; OV, ovarian cancer.

We also compared the somatic landscape and signatures of women who were carriers vs noncarriers of germline P/LP variants. Tumors that developed in carriers of HRR P/LP variants harbored more prevalent somatic *TP53* variants, regardless of cancer type, and generally had fewer variants in driver genes (eg, *PTEN*, *KRAS*, and *ERBB2* in ovarian cancer and *ARID1A* in both ovarian and endometrial cancer). The MMR germline carriers with endometrial cancer had more somatic MMR variants, while those with ovarian cancer harbored more common variants in driver genes, including *KRAS*, *ARID1A*, and *PTEN* (Figure 3A).

Germline P/LP variants in MMR genes (*MLH1*, *MSH2*, *MSH6*, and *PMS2*) were detected in 28.6% (4 of 14), 20.9% (9 of 43), and 14.3% (1 of 7) of high microsatellite instability (MSI-H) ovarian, endometrial, and cervical cancer, respectively (Figure 3B). Further analyses showed that 100% (3 of 3) of the biallelic inactivated ovarian cancers that developed in carriers of MMR P/LP variants had an MSI-H phenotype, while the proportion was 80.0% (8 of 10) in endometrial cancer. On the other hand, 1 of the 3 (33.3%) ovarian cancers and 1 of the 4 (25.0%) endometrial cancers that developed in carriers of MMR P/LP variants but retained the somatic heterozygosity of the germline-variant allele exhibited an MSI-H status (Figure 3C). Only 1 patient with cervical cancer harbored an MMR LP variant, and her tumor showed biallelic inactivation and an MSI-H status. The median tissue tumor mutational burden was generally higher in carriers of MMR P/LP variants but comparable in carriers of other germline variants compared with noncarriers across gynecologic cancer types (eFigure 4 in Supplement 1).

### Association of Germline P/LP Variants and Cancer Risk

We further investigated whether our germline findings were associated with an increased cancer risk using an East Asian population without cancer from gnomAD as the reference. In addition to well-known cancer-gene associations, we also identified that P/LP variants in *BRCA1* (OR, 5.47; 95% CI, 1.42-17.17; *P* = .008) and *FANCI* (OR, 5.53; 95% CI, 1.03-19.47; *P* = .02) were associated with endometrial cancer. The P/LP variants in *BRCA1* (OR, 4.92; 95% CI, 1.22-14.68; *P* = .01) and *BRCA2* (OR, 4.46; 95% CI, 1.11-13.14; *P* = .02) were associated with cervical cancer (Table 2).

**Table 2.** Cancer Predisposition of Germline Mutated Genes

Gene	Cancer altered, No.	Cancer total, No.	Control altered, No.	Control total, No.	Odds ratio (95% CI)	*P* value
**Ovarian cancer**						
*BRCA1*	110	945	21	9163	57.26 (35.47-96.28)	<.001
*BRCA2*	36	945	23	9097	15.61 (8.96-27.74)	<.001
*MLH1*	2	945	0	9012	∞ (1.79-∞)	.009
*MSH2*	4	945	3	9102	12.89 (2.18-88.04)	.002
*RAD51C*	7	945	16	8796	4.09 (1.42-10.55)	.005
*RAD51D*	14	945	19	8854	6.99 (3.23-14.75)	<.001
**Endometrial cancer**						
*MSH2*	9	307	3	9102	91.61 (22.66-526.57)	<.001
*MSH6*	6	307	10	9152	18.20 (5.40-55.61)	<.001
*MLH1*	2	307	0	9012	∞ (5.52-∞)	.001
*BRCA1*	4	307	21	9163	5.74 (1.42-17.17)	.008
*FANCI*	3	307	16	8990	5.53 (1.03-19.47)	.02
**Cervical cancer**						
*BRCA1*	4	358	21	9163	4.92 (1.22-14.68)	.01
*BRCA2*	4	358	23	9097	4.46 (1.11-13.14)	.02

## Discussion

In this cross-sectional study, we evaluated the prevalence and landscape of germline P/LP variants in unselected Chinese women with gynecologic cancers. Our results revealed a prevalence of 12.5% (202 of 1610) for germline carriers of variants in genes consistent with known tumor phenotypes and a prevalence of 3.7% (60 of 1610) for those with variants that are not known to be associated with the cancer. Cervical cancer has been thought to be a sporadic disease associated with genital infection of human papillomavirus and has little to do with rare inheritable variants. Our study identified a spectrum of deleterious germline variants, with a prevalence of 6.4% in cervical cancer. Most of these germline findings were in HRR genes, especially *BRCA1/2.* Interestingly, we found an association of HRR P/LP variants with earlier onset of cervical cancer, which often indicates an underlying inherited predisposition to cancer. Somatic biallelic inactivation observed in some of these HRR variants and the moderate cervical cancer risk of *BRCA1/2* P/LP variants further support that at least some of these germline findings may be driving events in cervical cancer. Nevertheless, the lower rate of biallelic inactivation (18.7%) compared with ovarian cancer (65.0%) also suggests that most of these P/LP variants may be incidental findings. Besides, a deleterious *MSH2* variant was identified in 1 patient with cervical cancer with a 2-hit event and MSI-H status, suggesting a role of this variant in the tumorigenesis of this case. Our results underscore the necessity of investigating inheritable factors in cervical cancer, which has long been neglected. Such studies may help to define a subgroup of patients with cervical cancer with distinctive molecular and clinicopathologic features who may benefit the most from germline testing.

For endometrial cancer, our study found a prevalence of HRR P/LP variants of 6.2%, comprising one-half of the germline findings in this disease. Notably, only a small proportion of these HRR variants occurred in *BRCA1/2*, with a prevalence of 2.0%. Previous studies also identified pathogenic *BRCA1/2* germline variants in 0.5% to 1.4% of patients with endometrial cancer.^23,24^ Based on the distinct clinicopathologic, molecular, and survival features of germline *BRCA1/2* variant carriers, another study suggested that endometrial cancer should be considered as one of the *BRCA1/2*-associated hereditary breast and ovarian cancer syndromes.^25^ A recent study reported a 2 to 3-fold increased risk for endometrial cancer in *BRCA1/2* variant carriers.^26^ We also observed that P/LP variants in *BRCA1* and *FANCI* were associated with a moderate risk for endometrial cancer, though a low rate of biallelic inactivation was observed. These results suggest potential involvement of pathogenic HRR germline variants in endometrial cancer development, which merits further investigation.

US Food and Drug Administration–approved targeted agents for managing gynecologic cancer remain limited.^27,28^ We found potential druggable germline variants in 15.3% of our cohort, comprising 95.1% of all germline findings. The majority of these variants were in HRR and MMR genes, suggesting therapeutic opportunities for poly(ADP-ribose) polymerase inhibitors and pembrolizumab. Moreover, prophylactic removal of both ovaries and fallopian tubes (salpingo-oophorectomy) and/or the uterus (hysterectomy) in *BRCA1/2* carriers is recognized as the most effective method for decreasing ovarian and endometrial cancer risk.^29^ Despite the substantial clinical relevance of germline findings in gynecologic cancer for therapeutic decision-making, genetic counseling, and intervention, current clinical guidelines only recommend germline testing for selected patients based on pathologic features of the tumor, onset age, and family history of cancer. The American Society of Clinical Oncology has recently recommended offering germline testing for women with epithelial ovarian cancer regardless of clinical features or family history.^30^ Germline testing of MMR genes is only recommended for patients with endometrial cancer whose tumors are MMR deficient or MSI-H, who have a family history of Lynch syndrome–related cancer, or who possess significant clinical characteristics for Lynch syndrome.^31^ Patients with cervical cancer are not referred for germline testing in the clinic. Notably, a large proportion of the clinically actionable germline findings stemming from our unselected cohort would have been missed if guideline-based genetic testing were offered. Despite the uncertain pathogenicity of some of these variants, identifying these germline findings could also benefit patients by providing implications for risk-reducing interventions and cancer surveillance and prevention measures in at-risk families.^32^

Emerging evidence suggests that germline variants, in addition to increasing cancer risk, also influence tumor progression by shaping the landscape of somatic alterations in cancer.^17,18^ Our study reveals some distinctive genomic features in cancers developing in carriers of pathogenic germline variants. Tumors in carriers of pathogenic HRR variants harbored more *TP53* variants than noncarriers, consistent with the finding that the loss of *TP53* function is associated with higher homologous recombination deficiency status.^33^ We also found that carriers of pathogenic HRR variants harbored fewer somatic variants in oncogenic driver genes than noncarriers. Consistently, Srinivasan et al^18^ recently showed the acquisition of fewer somatic oncogenic drivers as one of the hallmarks for tumors in carriers of germline variants in high-penetrance genes. Pathogenic germline variants can also increase the likelihood of somatic biallelic inactivation in the same gene or somatic alterations in the same pathway.^18,34^ Concordantly, we found that endometrial cancer developing in carriers of pathogenic MMR variants had higher somatic variant rates in the MMR genes. In contrast, MMR germline carriers with ovarian cancer showed higher somatic variant rates in oncogenic driver genes, suggesting a heterogeneous etiology of tumorigenesis mediated by MMR germline variants in endometrial cancer. Alternatively, these oncogenic variants may be just passenger events, given the higher mutation burden in these tumors.

### Limitations

This study had several limitations. The clinical significance of P/LP germline variants identified in our cohort lacks further validation, especially those without biallelic inactivation. Moreover, the targeted panel used in the study did not allow for the accurate determination of LOH status via a segment-based approach. Alternatively, we roughly estimated LOH status based on the mutation allele frequency (≥60%), assuming the tumor cell proportion to be 20% or greater (eMethods in Supplement 1). We had assessed this surrogate’s accuracy by using samples with a known LOH status determined by the standard segment-based approach. The cutoff of 60% resulted in a sensitivity of 65% and a specificity of 95%. Therefore, the LOH events may have been largely underestimated in our study. Moreover, the sample size for cervical cancer was small, which attenuates the strength of our findings on the associations of germline variants with age at onset and cancer risk.

## Conclusions

This study delineates the landscape of germline P/LP variants in cancer predisposition genes and reveals distinct somatic profiles in carriers of germline variants among unselected Chinese women with gynecologic cancers. The findings raise the importance of universal NGS-based genetic testing with a large gene panel as part of gynecologic cancer screening and diagnosis. Genetic testing could improve risk assessment, bring more opportunities for cancer prevention and early detection, and guide therapeutic decision-making.

## Notes

Supplement 1.
eMethods.

eReferences.
**eFigure 1.** Flow Diagram Illustrating the Cohort and Study Design **eFigure 2.** Comparison of 2-Hit Events for P/LP Variants vs Benign Variants **eFigure 3.** Comparison of the Germline P/LP Variant Rates Between Different Populations **eFigure 4.** Comparison of TMB Between Noncarriers (Germ–) and Carriers of Germline P/LP Variants in Different Gene Sets **eTable 1.** List of 62 Cancer-Predisposition Genes Included in the OncoScreen Plus Panel **eTable 2.** Detailed Information of Germline Pathogenic/Likely Pathogenic Variants Detected in the Cohort **eTable 3.** Distribution of Germline Pathogenic/Likely Pathogenic Variants Detected in the Cohort According to Genes and the Related Signaling Pathways **eTable 4.** Distribution of Germline Pathogenic/Likely Pathogenic Variants Detected from the Cohort According to Variant Type

Supplement 2.
Data Sharing Statement
