# Supplementary material for: Prevalence and Landscape of Pathogenic or Likely Pathogenic Germline Variants and Their Association With Somatic Phenotype in Unselected Chinese Patients With Gynecologic Cancers
Source: JAMA Netw Open. 2023 Jul 31;6(7):e2326437. doi: 10.1001/jamanetworkopen.2023.26437 (PMC10391307; doi:10.1001/jamanetworkopen.2023.26437)
Supplement: Supplement 1. — eMethods. eReferences. eFigure 1. Flow Diagram Illustrating the Cohort and Study Design eFigure 2. Comparison of 2-Hit Events for P/LP Variants vs Benign Variants eFigure 3. Comparison of the Germline P/LP Variant Rates Between Different Populations eFigure 4. Comparison of TMB Between Noncarriers (Germ–) and Carriers of Germline P/LP Variants in Different Gene Sets eTable 1. List of 62 Cancer-Predisposition Genes Included in the OncoScreen Plus Panel eTable 2. Detailed Information of Germline Pathogenic/Likely Pathogenic Variants Detected in the Cohort eTable 3. Distribution of Germline Pathogenic/Likely Pathogenic Variants Detected in the Cohort According to Genes and the Related Signaling Pathways eTable 4. Distribution of Germline Pathogenic/Likely Pathogenic Variants Detected from the Cohort According to Variant Type [file jamanetwopen-e2326437-s001.pdf]

Supplemental Online Content

Wen H, Xu Q, Sheng X, Li H, Wang X, Wu X. Prevalence and landscape of pathogenic or likely pathogenic germline variants and their association with somatic phenotype in unselected Chinese patients with gynecologic cancer. *JAMA Netw Open*. 2021;6(7):e2326437. doi:10.1001/jamanetworkopen.2023.26437

eMethods.

eReferences.

eFigure 1. Flow Diagram Illustrating the Cohort and Study Design

eFigure 2. Comparison of 2-Hit Events for P/LP Variants vs Benign Variants

eFigure 3. Comparison of the Germline P/LP Variant Rates Between Different Populations

eFigure 4. Comparison of TMB Between Noncarriers (Germ–) and Carriers of Germline P/LP Variants in Different Gene Sets

eTable 1. List of 62 Cancer-Predisposition Genes Included in the OncoScreen Plus Panel

eTable 2. Detailed Information of Germline Pathogenic/Likely Pathogenic Variants Detected in the Cohort

eTable 3. Distribution of Germline Pathogenic/Likely Pathogenic Variants Detected in the Cohort According to Genes and the Related Signaling Pathways

eTable 4. Distribution of Germline Pathogenic/Likely Pathogenic Variants Detected from the Cohort According to Variant Type

This supplemental material has been provided by the authors to give readers additional information about their work.

eMethods

Targeted next-generation sequencing (NGS)

Paired WBC and tumor samples were sequenced in Burning Rock Biotech, a clinical laboratory certified by the Clinical Laboratory Improvement Amendments (CLIA) and accredited by the College of American Pathologists (CAP). Germline mutations were profiled from genomic DNA extracted from blood samples. Somatic mutations were profiled from either tumor DNA extracted from formalin-fixed paraffin-embedded (FFPE) tissue or cytological specimen or circulating cell-free DNA extracted from liquid biopsy specimens. DNA samples were subjected to NGS library construction using optimized protocols as described previously [1]. Target capture was performed using a 520-gene panel (OncoScreen Plus, Burning Rock Biotech, Guangzhou, China). This panel also included 62 cancer predisposition genes, as listed in **Table S1**. These genes were selected based on the list of genes from the recommendation of reporting secondary findings of the American Society of Medical Genetics and Genomics (ACMG) [2], National Comprehensive Cancer Network (NCCN) Guidelines of Genetic/Familial High-Risk Assessment and Genomics guidelines [3]. Indexed samples were sequenced with paired-end reads using Nextseq 500 instrument (Illumina, CA, USA).

Classification of germline variants

The clinical significance of the variants was classified according to the ACMG guidelines for the interpretation and reporting of sequence variations as pathogenic (P), likely pathogenic (LP), variants of uncertain significance, likely benign, and benign. Only P and LP mutations were included in the subsequent analysis. The definition of therapeutically actionable mutations was adapted from [4].

Classification of somatic variants

© 2023 Wen H et al. *JAMA Network Open.*

The clinical significance of the somatic variants was categorized according to the recommendation of the Association for Molecular Pathology, American Society of Clinical Oncology, and CAP. Variants with strong clinical significance were classified as Tier I; variants with potential clinical significance as Tier II; variants of unknown clinical significance as Tier III; and variants deemed benign or likely benign as Tier IV [5]. Only variants categorized as Tiers I and II were included in the analysis.

Determination of loss of heterozygosity (LOH)

The status of LOH was evaluated for FFPE tissue samples only. If LOH of the germline mutated gene occurs, then the observed allele frequency (AF) for the germline mutation is 100x0.2+50x0.8=60% when the tumor cell proportion is 20%. LOH of the germline mutated gene was determined as positive for a given sample if the AF of the germline mutation was ⩾60%, assuming the tumor cell proportion⩾20%.

Tumor mutation burden (TMB) calculation

TMB was calculated for a given sample as the ratio between the number of detected somatic mutations with the total size (1.003 Mb) of the coding region of the 520 gene panel using the formula below. The mutation count included non-synonymous single nucleotide variants (SNVs) and small insertion-deletion variants (Indels) detected within the coding region and ±2bp upstream or downstream region and does not include hot mutation events, copy number variants (CNVs), structural variants (SVs), and germline SNPs. Only mutations with allelic fraction (AF) of ≥ 2% for tissue samples and ≥ 0.2% for liquid biopsy samples were included in the mutation count. For accurate TMB calculation, maximum AF (maxAF) should be ≥ 5% for tissue samples and ≥ 1% for liquid biopsy samples.

$$\mathrm{TMB} = \frac{\text{mutation count (except for CNV, SV, SNPs, and hot mutations)}}{1.003\ \mathrm{Mb}}$$

Determination of microsatellite instability (MSI) status

© 2023 Wen H et al. *JAMA Network Open.*

The MSI status was determined based on a read-count distribution approach as previously described [6,7]. Briefly, a total of 63 microsatellite loci were selected for categorization. The coverage ratio of a specific set of repeat lengths was calculated for each locus, and the locus was categorized as unstable if the coverage ratio was less than [mean − 3 × SD] of the reference ratio. A given sample was determined as MSI-high (MSI-H) if more than 40% of the microsatellite loci were length-instable, MSI-low (MSI-L) if the percentage of length-instable loci was between 15% and 40%, or microsatellite stability (MSS) if the percentage were <15%.[1].

eReferences

1. Wang M, Chen X, Dai Y, et al. Concordance Study of a 520-Gene Next-Generation Sequencing-Based Genomic Profiling Assay of Tissue and Plasma Samples. *Mol Diagn Ther*. Mar 19 2022;doi:10.1007/s40291-022-00579-1
2. Richards S, Aziz N, Bale S, et al. Standards and guidelines for the interpretation of sequence variants: a joint consensus recommendation of the American College of Medical Genetics and Genomics and the Association for Molecular Pathology. *Genet Med*. May 2015;17(5):405-24. doi:10.1038/gim.2015.30
3. Daly MB, Pilarski R, Yurgelun MB, et al. NCCN Guidelines Insights: Genetic/Familial High-Risk Assessment: Breast, Ovarian, and Pancreatic, Version 1.2020. *J Natl Compr Canc Netw*. Apr 2020;18(4):380-391. doi:10.6004/jnccn.2020.0017
4. Thavaneswaran S, Rath E, Tucker K, et al. Therapeutic implications of germline genetic findings in cancer. *Nat Rev Clin Oncol*. Jun 2019;16(6):386-396. doi:10.1038/s41571-019-0179-3
5. Li MM, Datto M, Duncavage EJ, et al. Standards and Guidelines for the Interpretation and Reporting of Sequence Variants in Cancer: A Joint Consensus Recommendation of the Association for Molecular Pathology, American Society of Clinical Oncology, and College of American Pathologists. *J Mol Diagn*. Jan 2017;19(1):4-23. doi:10.1016/j.jmoldx.2016.10.002
6. Zhu L, Huang Y, Fang X, et al. A Novel and Reliable Method to Detect Microsatellite Instability in Colorectal Cancer by Next-Generation Sequencing. *J Mol Diagn*. Mar 2018;20(2):225-231. doi:10.1016/j.jmoldx.2017.11.007
7. Cai Z, Wang Z, Liu C, et al. Detection of Microsatellite Instability from Circulating Tumor DNA by Targeted Deep Sequencing. *J Mol Diagn*. Jul 2020;22(7):860-870. doi:10.1016/j.jmoldx.2020.04.210

© 2023 Wen H et al. *JAMA Network Open.*

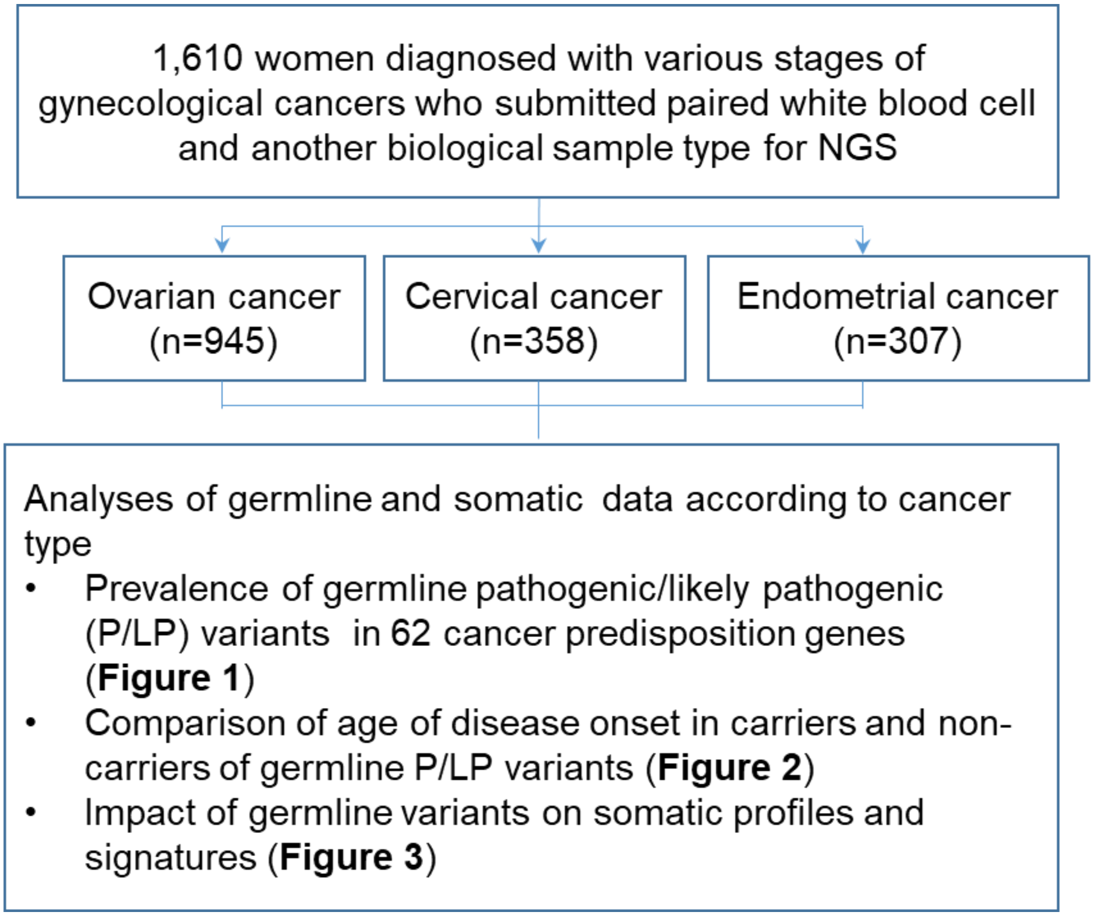

eFigure 1. Flow Diagram Illustrating the Cohort and Study Design

© 2023 Wen H et al. *JAMA Network Open.*

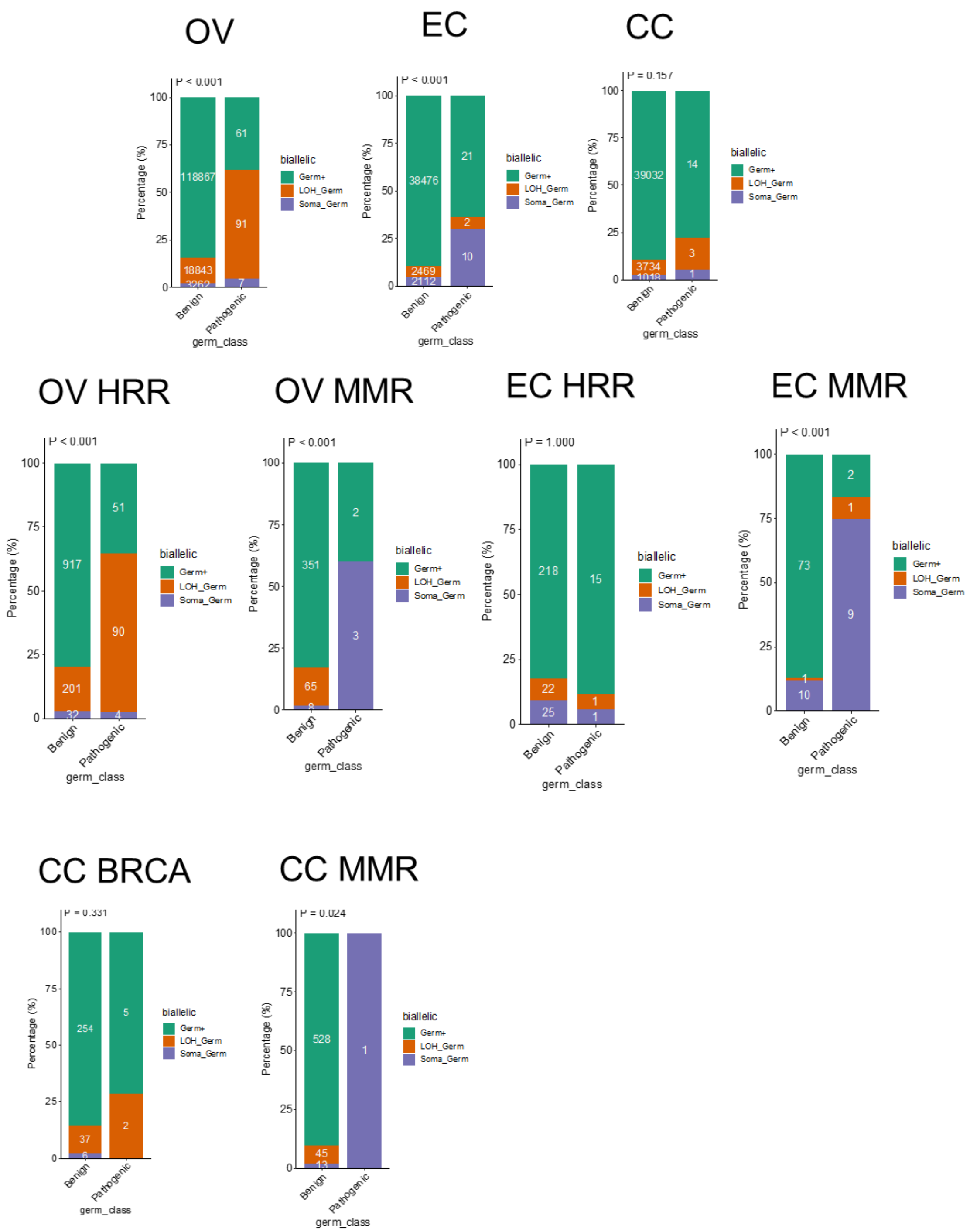

eFigure 2. Comparison of 2-Hit Events for P/LP Variants vs Benign Variants. OV: ovarian cancer; EC: endometrial cancer; CC: cervical cancer.

© 2023 Wen H et al. *JAMA Network Open.*

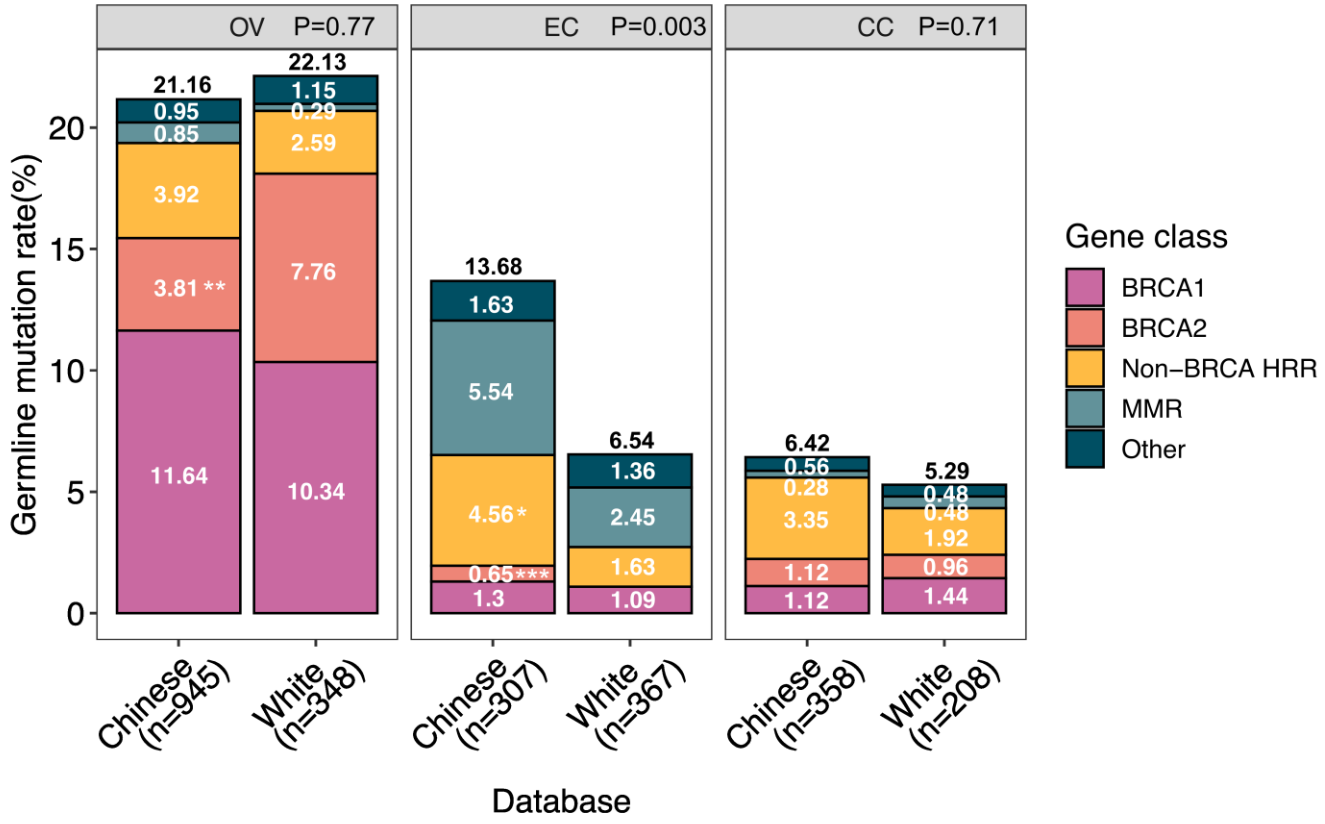

eFigure 3. Comparison of the Germline P/LP Variant Rates Between Different Populations. Comparison of the germline P/LP variant rates among different genes between our cohort (Chinese) and TCGA White cohort (doi:10.1016/j.cell.2018.03.039). Note: asterisks represent the level of statistical significance wherein * represents p<0.05, ** represents p<0.01, *** represents p<0.001.

© 2023 Wen H et al. *JAMA Network Open.*

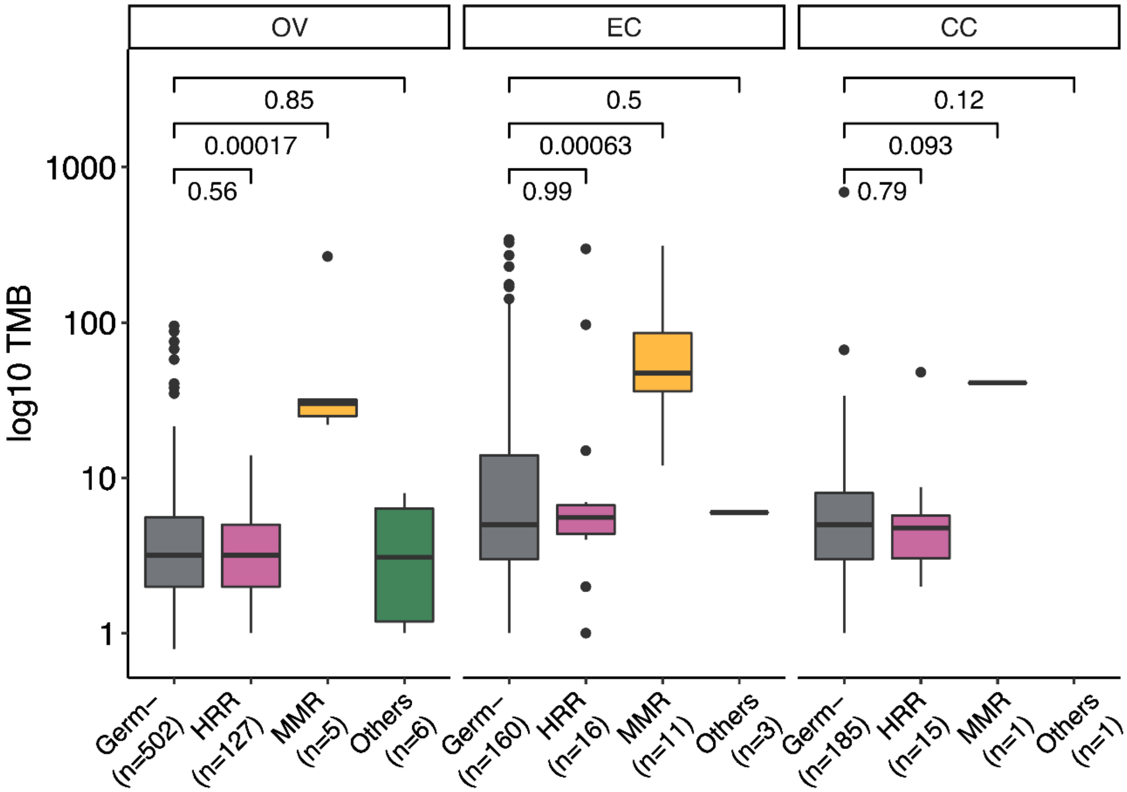

eFigure 4. Comparison of TMB Between Noncarriers (Germ–) and Carriers of Germline P/LP Variants in Different Gene Sets

© 2023 Wen H et al. *JAMA Network Open.*

eTable 1. List of 62 Cancer-Predisposition Genes Included in the OncoScreen Plus Panel

Genes			
APC	*FANCA*	***NF1***	***RAD54L***
ATM	***FANCI***	*NF2*	*RB1*
AXIN2	***FANCL***	*NTHL1*	***RET***
BARD1	*FH*	***PALB2***	*SDHA*
BMPR1A	*FLCN*	*PDGFRA*	*SDHAF2*
BRCA1	*GREM1*	***PMS2***	*SDHB*
BRCA2	*HOXB13*	*POLD1*	*SDHC*
BRIP1	***KIT***	*POLE*	*SDHD*
CDH1	*MEN1*	*PPP2R2A*	*SMAD4*
CDK12	***MET***	*PRKAR1A*	*STK11*
CDK4	***MLH1***	***PTCH1***	*TP53*
CDKN2A	***MSH2***	*PTEN*	***TSC1***
CHEK1	*MSH3*	***RAD51B***	***TSC2***
CHEK2	***MSH6***	***RAD51C***	*VHL*
EGFR	*MUTYH*	***RAD51D***	*WT1*
EPCAM	*NBN*		

Note: the 28 gene names in boldface were considered actionable genes

© 2023 Wen H et al. *JAMA Network Open.*

eTable 2. Detailed Information of Germline Pathogenic/Likely Pathogenic Variants Detected in the Cohort

Patient	Age	Cancer	Gene	Variant type	Description	hgvs_p	hgvs_c	AF	Class
P1	67	OV	BRCA2	frameshift_variant	p.V220fs	p.Val220fs	c.658_659del	51.53%	P
P10	45	OV	MSH2	frameshift_variant	p.L478fs	p.Leu478fs	c.1433_1434del	42.75%	LP
P100		OV	BRCA1	missense_variant	p.A1752P	p.Ala1752Pro	c.5254G>C	79.87%	LP
P101		OV	BRCA1	frameshift_variant	p.N704fs	p.Asn704fs	c.2110_2111del	77.65%	P
P102		OV	BRCA2	frameshift_variant	p.N1055fs	p.Asn1055fs	c.3165_3168del	86.55%	LP
P103		OV	BRCA1	frameshift_variant	p.I1824fs	p.Ile1824fs	c.5470_5477del	50.00%	P
P104		OV	SDHA	start_lost	p.M1?	p.Met1?	c.1A>G	45.71%	P
P105		OV	BRCA2	frameshift_variant	p.E2183fs	p.Glu2183fs	c.6547del	70.71%	LP
P106		OV	BRCA1	frameshift_variant	p.Q1111fs	p.Gln1111fs	c.3329dup	63.58%	P
P107		OV	BRCA1	frameshift_variant	p.E1257fs	p.Glu1257fs	c.3770_3771del	94.13%	P
P108		OV	RAD51D	stop_gained	p.R300*	p.Arg300*	c.898C>T	98.32%	P
P109		OV	BRCA1	frameshift_variant	p.N537fs	p.Asn537fs	c.1608del	86.49%	LP
P111	61	OV	BRCA1	frameshift_variant	p.E1257fs	p.Glu1257fs	c.3770_3771del	67.07%	P
P112	37	OV	BRCA1	splice_region_variant	c.213-12A>G		c.213-12A>G	58.59%	P
P113	47	OV	BRCA1	frameshift_variant	p.N665fs	p.Asn665fs	c.1994del	64.82%	P
P114	52	OV	RAD51D	frameshift_variant	p.K91fs	p.Lys91fs	c.270_271dup	79.94%	P
P115	42	OV	BRCA1	stop_gained	p.S713*	p.Ser713*	c.2138C>G	54.39%	P
			FANCA	splice_acceptor_variant	c.4011-1G>A		c.4011-1G>A	45.50%	LP
P116	57	OV	BRCA1	frameshift_variant	p.E23fs	p.Glu23fs	c.66dup	50.00%	P
P117	47	OV	MUTYH	stop_gained	p.W156*	p.Trp156*	c.467G>A	47.73%	P
P118	64	OV	RAD51D	frameshift_variant	p.K91fs	p.Lys91fs	c.270_271dup	62.58%	P
P119	54	OV	BRCA2	frameshift_variant	p.L2523fs	p.Leu2523fs	c.7567_7568del	68.43%	P
P12	47	OV	BRCA1	missense_variant	p.G1743E	p.Gly1743Glu	c.5228G>A	44.56%	LP
P120	65	OV	CHEK2	stop_gained	p.S33*	p.Ser33*	c.98C>G	71.18%	LP
P121	74	OV	FANCI	stop_gained	p.L160*	p.Leu160*	c.479T>A	72.43%	LP
P123	74	OV	MUTYH	stop_gained	p.Q267*	p.Gln267*	c.799C>T	50.23%	P
			SDHA	start_lost	p.M1?	p.Met1?	c.2T>C	23.96%	LP
P124	53	OV	BRCA1	splice_region_variant	c.80+5G>A		c.80+5G>A	48.88%	P
P125	59	OV	BRCA2	frameshift_variant	p.N1287fs	p.Asn1287fs	c.3860del	89.31%	P
P126	44	OV	BRCA1	frameshift_variant	p.N1355fs	p.Asn1355fs	c.4065_4068del	74.06%	P

© 2023 Wen H et al. *JAMA Network Open.*

P127	53	OV	BRCA1	frameshift_variant	p.I1824fs	p.Ile1824fs	c.5470_5477del	71.00%	P
P128	56	OV	MSH6	frameshift_variant	p.I1239fs	p.Ile1239fs	c.3716_3717del	46.99%	P
P129	61	OV	BRCA1	stop_gained	p.S573*	p.Ser573*	c.1718C>A	53.09%	LP
P13	50	OV	BRCA1	frameshift_variant	p.K1095fs	p.Lys1095fs	c.3285del	47.45%	P
P131	32	OV	BRCA1	missense_variant		p.Leu95Pro	c.284T>C	52.35%	LP
P132	41	OV	BRCA1	stop_gained	p.K338*	p.Lys338*	c.1012A>T	79.23%	P
P133	56	OV	BRCA1	missense_variant	p.C1697R	p.Cys1697Arg	c.5089T>C	50.36%	LP
P134	69	OV	NBN	frameshift_variant	p.P433fs	p.Pro433fs	c.1298del	65.72%	LP
P135	74	OV	MSH3	frameshift_variant	p.I859fs	p.Ile859fs	c.2575del	47.98%	LP
P136	32	OV	BRCA1	stop_gained	p.E489*	p.Glu489*	c.1465G>T	67.62%	P
P137	46	OV	BRCA1	frameshift_variant	p.N1647fs	p.Asn1647fs	c.4941del	51.42%	P
P138	50	OV	BRCA1	frameshift_variant	p.Y655fs	p.Tyr655fs	c.1961dup	68.41%	P
P139	43	OV	BRCA1	splice_donor_variant	c.4185+1G>A		c.4185+1G>A	48.39%	P
P14	48	OV	BRCA2	frameshift_variant	p.K2162fs	p.Lys2162fs	c.6486_6489del	38.90%	P
P140	57	OV	BRCA1	missense_variant	p.L1786P	p.Leu1786Pro	c.5357T>C	51.92%	LP
P141	52	OV	BRCA2	frameshift_variant	p.T630fs	p.Thr630fs	c.1888dup	86.28%	P
P142	44	OV	BRCA1	stop_gained	p.R1835*	p.Arg1835*	c.5503C>T	84.17%	P
P143	72	OV	BRIP1	large_genomic_rearrangement	exon5-6cn_del		exon5-6cn_del	0.9	LP
P144	65	OV	BRCA1	large_genomic_rearrangement	exon17-18cn_del			1.1	LP
P145	57	OV	BRCA1	missense_variant	p.Y1703C	p.Tyr1703Cys	c.5108A>G	26.62%	LP
P146	42	OV	BARD1	stop_gained	p.S376*	p.Ser376*	c.1127C>G	48.75%	LP
P148	70	OV	BRIP1	frameshift_variant	p.K567fs	p.Lys567fs	c.1695_1699delinsTGAC	56.33%	LP
P15	54	OV	BRCA2	frameshift_variant	p.R858fs	p.Arg858fs	c.2570dup	50.40%	P
P152	51	OV	BRCA1	frameshift_variant	p.E23fs	p.Glu23fs	c.66dup	73.88%	P
P153	52	OV	RAD51D	frameshift_variant	p.K91fs	p.Lys91fs	c.270_271dup	47.54%	P
P154	54	OV	MSH3	stop_gained	p.Q242*	p.Gln242*	c.724C>T	45.97%	LP
			BRIP1	stop_gained	p.W468*	p.Trp468*	c.1403G>A	50.04%	LP
P156	49	OV	BRCA1	frameshift_variant	p.V923fs	p.Val923fs	c.2767_2770del	74.71%	P
P159	62	OV	PALB2	stop_gained	p.S70*	p.Ser70*	c.209C>A	79.06%	LP
P16	64	OV	BRCA1	frameshift_variant	p.N1355fs	p.Asn1355fs	c.4065_4068del	49.78%	P
P161	66	OV	BRIP1	frameshift_variant	p.E501fs	p.Glu501fs	c.1500dup	76.41%	LP
P162	38	OV	BRCA1	stop_gained	p.Q1458*	p.Gln1458*	c.4372C>T	88.93%	P

© 2023 Wen H et al. *JAMA Network Open.*

P163	71	OV	BRCA1	stop_gained	p.R1443*	p.Arg1443*	c.4327C>T	51.59%	P
P164	29	OV	ATM	stop_gained	p.Q1310*	p.Gln1310*	c.3928C>T	53.54%	LP
P165	52	OV	BRCA2	frameshift_variant	p.E2183fs	p.Glu2183fs	c.6547del	88.16%	LP
P166	54	OV	RAD51D	frameshift_variant	p.K91fs	p.Lys91fs	c.270_271dup	40.35%	P
P167	49	OV	BRCA1	frameshift_variant	p.I1824fs	p.Ile1824fs	c.5470_5477del	61.48%	P
P168	40	OV	BRCA2	frameshift_variant	p.E2558fs	p.Glu2558fs	c.7673_7674del	66.83%	P
P169	68	OV	RAD51D	frameshift_variant	p.K91fs	p.Lys91fs	c.270_271dup	83.33%	P
P170	52	OV	BRCA1	frameshift_variant	p.I1824fs	p.Ile1824fs	c.5470_5477del	91.16%	P
P171	50	OV	BRCA2	frameshift_variant	p.N2135fs	p.Asn2135fs	c.6405_6409del	84.66%	P
P172	49	OV	BRCA1	splice_region_variant			c.5074+3A>G	70.88%	LP
P174	61	OV	BRCA2	stop_gained	p.Q1063*	p.Gln1063*	c.3187C>T	74.44%	P
P176	69	OV	BRCA1	frameshift_variant	p.C328fs	p.Cys328fs	c.981_982del	59.18%	P
P177	51	OV	BRCA1	large_genomic_rearrangement	exon16-19cn_del			0.4	LP
P178	56	OV	BRCA1	stop_gained	p.E1158*	p.Glu1158*	c.3472G>T	86.64%	P
P179	63	OV	BRCA1	large_genomic_rearrangement	exon2-21del		exon2-21del	1	LP
P18	33	OV	MLH1	frameshift_variant	p.V752fs	p.Val752fs	c.2252_2253dup	50.06%	LP
P181	51	OV	BRCA1	splice_region_variant	p.C44=	p.Cys44=	c.132C>T	62.12%	LP
P182	50	OV	BRCA1	frameshift_variant	p.G964fs	p.Gly964fs	c.2889_2890del	11.10%	P
P184	53	OV	BRCA1	missense_variant	p.T1691K	p.Thr1691Lys	c.5072C>A	82.06%	LP
			FANCA	splice_region_variant	c.709+5G>A		c.709+5G>A	45.76%	P
P186		OV	BRIP1	frameshift_variant	p.S1025fs	p.Ser1025fs	c.3072del	80.71%	LP
			BRCA2	frameshift_variant	p.K437fs	p.Lys437fs	c.1310_1313del	70.73%	P
P187	70	OV	BRCA2	splice_acceptor_variant	c.-39-1_-39del		c.-39-1_-39del	48.31%	P
P189	52	OV	BRCA1	stop_gained	p.S361*	p.Ser361*	c.1082C>G	89.89%	P
P190	63	OV	RAD51D	frameshift_variant	p.K91fs	p.Lys91fs	c.270_271dup	55.44%	P
P191	44	OV	BRCA1	stop_gained	p.E1158*	p.Glu1158*	c.3472G>T	24.09%	P
P192	54	OV	RAD51C	splice_acceptor_variant	c.905-2A>C		c.905-2A>C	60.23%	P
P193	44	OV	BRCA1	frameshift_variant	p.E1148fs	p.Glu1148fs	c.3442del	80.83%	P
P194	69	OV	BRCA1	frameshift_variant	p.T293fs	p.Thr293fs	c.876_879del	50.50%	P
P195	60	OV	BRCA2	frameshift_variant	p.Y3092fs	p.Tyr3092fs	c.9275_9276del	89.12%	P
P196	56	OV	BRCA1	stop_gained	p.E879*	p.Glu879*	c.2635G>T	84.54%	P
P197	63	OV	BRCA1	frameshift_variant	p.T1163fs	p.Thr1163fs	c.3487dup	57.63%	LP

© 2023 Wen H et al. *JAMA Network Open.*

P198	57	OV	BRCA2	frameshift_variant	p.S1943fs	p.Ser1943fs	c.5826_5827del	52.64%	P
P199	62	OV	RAD51D	frameshift_variant	p.K91fs	p.Lys91fs	c.270_271dup	59.00%	P
P2	50	OV	BRCA1	stop_gained	p.K338*	p.Lys338*	c.1012A>T	56.39%	P
P201	50	OV	BRCA2	frameshift_variant	p.L1908fs	p.Leu1908fs	c.5722_5723del	87.79%	P
P203	67	OV	BRCA1	missense_variant		p.Phe1695Val	c.5083T>G	86.20%	LP
P206	59	OV	RAD51C	splice_acceptor_variant	c.905-2_905-1del		c.905-2_905-1del	67.78%	P
P207	53	OV	BRCA1	frameshift_variant	p.I1824fs	p.Ile1824fs	c.5470_5477del	73.50%	P
P208	58	OV	RAD51D	stop_gained	p.R186*	p.Arg186*	c.556C>T	60.17%	P
P209	47	OV	BRCA1	stop_gained	p.Y1429*	p.Tyr1429*	c.4287C>A	46.72%	P
P21	56	OV	BRCA1	frameshift_variant	p.E1210fs	p.Glu1210fs	c.3627dup	48.20%	P
P210	37	OV	MLH1	intron_variant	c.454-13A>G		c.454-13A>G	50.11%	P
P211	62	OV	BRCA1	frameshift_variant	p.E720fs	p.Glu720fs	c.2157dup	38.54%	P
P212	47	OV	BRCA1	frameshift_variant	p.T97fs	p.Thr97fs	c.290_291del	48.22%	P
P214	46	OV	RAD51C	large_genomic_rearrangement	exon6-9del		exon6-9del	0.7	LP
P215		OV	BRCA1	missense_variant		p.Trp1718Arg	c.5152T>C	58.24%	LP
P217	46	OV	PMS2	frameshift_variant	p.L236fs	p.Leu236fs	c.707del	25.09%	LP
P218	53	OV	BRCA1	frameshift_variant	p.E1148fs	p.Glu1148fs	c.3442del	88.52%	P
P219	58	OV	FANCI	splice_donor_variant	c.1698+1G>A		c.1698+1G>A	49.12%	LP
P220	49	OV	BRCA2	splice_region_variant	p.P3039=	p.Pro3039=	c.9117G>A	50.28%	P
P222	46	OV	BRCA1	missense_variant	p.V1714G	p.Val1714Gly	c.5141T>G	47.66%	LP
P223	56	OV	BRCA1	frameshift_variant	p.P1099fs	p.Pro1099fs	c.3291_3294del	40.11%	LP
P224	58	OV	BRCA1	stop_gained	p.K1601*	p.Lys1601*	c.4801A>T	49.39%	P
P225	56	OV	BRCA2	frameshift_variant	p.N2135fs	p.Asn2135fs	c.6405_6409del	63.62%	P
P226	52	OV	BRCA1	frameshift_variant	p.S308fs	p.Ser308fs	c.922_924delinsT	66.90%	P
P228	44	OV	BRCA1	stop_gained	p.E418*	p.Glu418*	c.1252G>T	85.93%	P
P229	56	OV	BRCA2	frameshift_variant	p.H1332fs	p.His1332fs	c.3993del	83.81%	LP
P23	55	OV	BRCA2	stop_gained	p.S1882*	p.Ser1882*	c.5645C>A	40.68%	P
P230	34	OV	RET	missense_variant	p.R886W	p.Arg886Trp	c.2656C>T	32.56%	LP
P232	56	OV	BRCA1	frameshift_variant	p.I1824fs	p.Ile1824fs	c.5470_5477del	78.07%	P
P233	38	OV	BRCA1	frameshift_variant	p.F1571fs	p.Phe1571fs	c.4712_4716del	85.48%	P
P234	61	OV	BRCA1	stop_gained	p.W353*	p.Trp353*	c.1058G>A	45.41%	P
P235	57	OV	BRCA1	missense_variant	p.T1691K	p.Thr1691Lys	c.5072C>A	90.24%	LP

© 2023 Wen H et al. *JAMA Network Open.*

P237	44	OV	RAD51C	stop_gained	p.G130*	p.Gly130*	c.388G>T	45.74%	LP
P238	54	OV	BRCA1	frameshift_variant		p.Glu1112fs	c.3333del	76.08%	P
P239		OV	BRCA1	missense_variant	p.C64W	p.Cys64Trp	c.192T>G	75.72%	LP
P24	47	OV	RAD51D	frameshift_variant	p.K91fs	p.Lys91fs	c.270_271dup	70.67%	P
P240	51	OV	BRCA2	frameshift_variant	p.N2135fs	p.Asn2135fs	c.6405_6409del	89.57%	P
P241	75	OV	BRCA1	missense_variant	p.C39Y	p.Cys39Tyr	c.116G>A	52.77%	LP
P243	62	OV	BRCA2	stop_gained	p.Q321*	p.Gln321*	c.961C>T	48.77%	P
P244	47	OV	MSH2	missense_variant	p.L173R	p.Leu173Arg	c.518T>G	47.81%	LP
P245	57	OV	BRCA1	missense_variant		p.Cys1697Tyr	c.5090G>A	52.93%	LP
P247	44	OV	BRCA1	splice_donor_variant	c.4185+1G>A		c.4185+1G>A	47.35%	P
P248	54	OV	BRCA2	stop_gained	p.Q1037*	p.Gln1037*	c.3109C>T	55.62%	P
P25	53	OV	BRCA1	stop_gained	p.R1203*	p.Arg1203*	c.3607C>T	52.63%	P
P250	61	OV	RAD51D	stop_gained	p.R300*	p.Arg300*	c.898C>T	73.29%	P
			BRCA2	frameshift_variant	p.G1122fs	p.Gly1122fs	c.3365del	50.08%	P
P251	56	OV	BRCA1	frameshift_variant	p.R1076fs	p.Arg1076fs	c.3226del	49.38%	P
P254	57	OV	BRCA1	frameshift_variant	p.G1077fs	p.Gly1077fs	c.3228_3229del	76.79%	P
P255	58	OV	BRCA1	frameshift_variant	p.Q1111fs	p.Gln1111fs	c.3329dup	75.31%	P
P256	53	OV	BRCA1	frameshift_variant	p.P1099fs	p.Pro1099fs	c.3294del	73.52%	P
P257	68	OV	BRCA2	missense_variant	p.E3002K	p.Glu3002Lys	c.9004G>A	53.55%	LP
P258	39	OV	FANCA	stop_gained	p.R591*	p.Arg591*	c.1771C>T	47.52%	P
P26	50	OV	RAD51C	stop_gained	p.E334*	p.Glu334*	c.1000G>T	43.08%	LP
P28	37	OV	BRCA1	frameshift_variant	p.F1571fs	p.Phe1571fs	c.4712_4716del	43.04%	P
P29	73	OV	BRCA1	stop_gained	p.R1443*	p.Arg1443*	c.4327C>T	90.10%	P
P3	37	OV	BRCA1	stop_gained	p.K1601*	p.Lys1601*	c.4801A>T	82.09%	P
P30	62	OV	BRCA1	stop_gained	p.K1601*	p.Lys1601*	c.4801A>T	49.53%	P
P31	71	OV	BRCA2	frameshift_variant	p.N2460fs	p.Asn2460fs	c.7379_7382del	62.48%	P
P33	44	OV	MSH2	stop_gained	p.R383*	p.Arg383*	c.1147C>T	50.50%	P
P34	55	OV	BRCA1	frameshift_variant	p.S1841fs	p.Ser1841fs	c.5521del	85.39%	P
P35	52	OV	BRCA1	frameshift_variant	p.K935fs	p.Lys935fs	c.2804_2807del	61.26%	LP
P37	50	OV	BRCA2	stop_gained	p.Q1037*	p.Gln1037*	c.3109C>T	49.43%	P
P4	55	OV	BRCA2	frameshift_variant	p.H2147fs	p.His2147fs	c.6440_6443del	47.91%	LP
P40	63	OV	BRCA2	frameshift_variant	p.N986fs	p.Asn986fs	c.2957dup	45.54%	P

© 2023 Wen H et al. *JAMA Network Open.*

P41	62	OV	BRCA1	frameshift_variant	p.S157fs	p.Ser157fs	c.470_471del	80.65%	P
P42	56	OV	BRCA1	stop_gained	p.Q1090*	p.Gln1090*	c.3268C>T	49.23%	P
P43	58	OV	BRCA1	splice_acceptor_variant	c.81-2A>G		c.81-2A>G	52.78%	P
P48	48	OV	MUTYH	stop_gained	p.W156*	p.Trp156*	c.467G>A	48.25%	P
P49	77	OV	RAD51D	stop_gained	p.R300*	p.Arg300*	c.898C>T	49.16%	P
P5	68	OV	BRCA1	frameshift_variant	p.A1206fs	p.Ala1206fs	c.3612_3613del	43.23%	LP
P50	36	OV	BRCA1	frameshift_variant	p.L502fs	p.Leu502fs	c.1504_1508del	45.05%	P
P52	49	OV	BRCA1	frameshift_variant	p.P968fs	p.Pro968fs	c.2903del	90.38%	LP
P53	44	OV	BRCA1	frameshift_variant	p.E1115fs	p.Glu1115fs	c.3342_3345del	67.33%	P
P56	44	OV	BRCA1	stop_gained	p.R1443*	p.Arg1443*	c.4327C>T	52.67%	P
P57	56	OV	BRCA1	stop_gained	p.Q1525*	p.Gln1525*	c.4573C>T	56.87%	P
P60	34	OV	BRCA1	frameshift_variant	p.E23fs	p.Glu23fs	c.66dup	72.10%	P
P61	54	OV	BRCA1	frameshift_variant	p.L1153fs	p.Leu1153fs	c.3458del	45.96%	LP
P63	58	OV	BRCA1	stop_gained	p.R1203*	p.Arg1203*	c.3607C>T	82.63%	P
P64	57	OV	BRCA1	splice_donor_variant	c.5406+1_5406+3del		c.5406+1_5406+3del	45.41%	LP
P67	46	OV	RAD51C	splice_acceptor_variant	c.1027-1G>T		c.1027-1G>T	58.45%	LP
P69	67	OV	RAD51D	frameshift_variant	p.K91fs	p.Lys91fs	c.270_271dup	65.47%	P
P7	66	OV	BRCA1	frameshift_variant	p.Y655fs	p.Tyr655fs	c.1961dup	40.31%	P
P70	57	OV	BRCA1	frameshift_variant	p.S1841fs	p.Ser1841fs	c.5521del	51.22%	P
P71	59	OV	BRCA1	frameshift_variant	p.K428fs	p.Lys428fs	c.1281_1312delinsATATGT	36.93%	LP
P72	57	OV	RAD51C	frameshift_variant	p.F148fs	p.Phe148fs	c.444del	61.91%	LP
P73	66	OV	BRCA1	splice_region_variant			c.212+3A>T	50.48%	LP
P76	54	OV	MSH2	splice_region_variant	c.942+3A>T		c.942+3A>T	23.60%	P
P77	68	OV	BRCA2	frameshift_variant	p.T1553fs	p.Thr1553fs	c.4657_4658del	43.40%	LP
P78	54	OV	BRCA2	frameshift_variant	p.L2039fs	p.Leu2039fs	c.6116del	48.57%	LP
P79	55	OV	ATM	frameshift_variant	p.F407fs	p.Phe407fs	c.1221del	48.12%	LP
P81	48	OV	BRCA1	stop_gained	p.R1203*	p.Arg1203*	c.3607C>T	54.05%	P
P83	57	OV	BRCA1	frameshift_variant	p.E1115fs	p.Glu1115fs	c.3342_3345del	82.50%	P
P84	42	OV	BRCA1	frameshift_variant	p.E1115fs	p.Glu1115fs	c.3342_3345del	45.94%	P
P86	34	OV	BRCA1	frameshift_variant	p.P1150fs	p.Pro1150fs	c.3449del	79.76%	LP
P88	50	OV	BRCA1	frameshift_variant	p.L290fs	p.Leu290fs	c.869del	70.12%	LP
P89		OV	BRCA2	frameshift_variant	p.E260fs	p.Glu260fs	c.774_775del	84.15%	P

© 2023 Wen H et al. *JAMA Network Open.*

P9	46	OV	BRCA2	stop_gained	p.Y1762*	p.Tyr1762*	c.5286T>G	79.65%	LP
P91	61	OV	RAD51D	frameshift_variant	p.K91fs	p.Lys91fs	c.270_271dup	45.91%	P
P92	53	OV	BRCA1	frameshift_variant	p.A322fs	p.Ala322fs	c.964del	63.14%	P
P93	40	OV	BRCA1	frameshift_variant	p.C328fs	p.Cys328fs	c.981_982del	81.64%	P
P94	56	OV	BRCA2	stop_gained	p.Q1295*	p.Gln1295*	c.3883C>T	46.67%	P
P95		OV	BRCA2	stop_gained	p.Y688*	p.Tyr688*	c.2064T>A	80.82%	LP
P96		OV	PALB2	frameshift_variant		p.Lys346fs	c.1037_1041del	36.51%	P
P97		OV	BRCA1	frameshift_variant	p.I1824fs	p.Ile1824fs	c.5470_5477del	86.80%	P
P98		OV	BRCA1	frameshift_variant	p.I1061fs	p.Ile1061fs	c.3181del	82.75%	P
P99	54	OV	BRCA1	stop_gained	p.E1114*	p.Glu1114*	c.3340G>T	72.99%	P
P11	65	EC	MSH6	frameshift_variant	p.T1225fs	p.Thr1225fs	c.3674del	43.44%	LP
P130	58	EC	MSH6	stop_gained	p.R1068*	p.Arg1068*	c.3202C>T	42.41%	P
P149	27	EC	MLH1	missense_variant	p.A21V	p.Ala21Val	c.62C>T	98.07%	LP
P150	57	EC	RAD54L	splice_acceptor_variant	c.408-2A>G		c.408-2A>G	50.21%	LP
P160	86	EC	FANCI	stop_gained	p.Y1210*	p.Tyr1210*	c.3630T>G	44.64%	LP
P17	70	EC	RAD54L	splice_donor_variant	c.3+1G>C		c.3+1G>C	56.66%	LP
P173	77	EC	FANCI	stop_gained	p.R1299*	p.Arg1299*	c.3895C>T	45.63%	LP
P175	38	EC	MSH2	stop_gained	p.Q170*	p.Gln170*	c.508C>T	40.02%	P
P183	54	EC	MSH2	stop_gained	p.Y405*	p.Tyr405*	c.1215C>A	47.44%	P
P185	55	EC	MSH2	frameshift_variant	p.N486fs	p.Asn486fs	c.1457_1460del	46.42%	P
P188	65	EC	MUTYH	missense_variant	p.G286E	p.Gly286Glu	c.857G>A	49.15%	LP
P19	52	EC	BRCA2	stop_gained	p.E1455*	p.Glu1455*	c.4363G>T	48.55%	LP
P20	53	EC	BRCA1	frameshift_variant	p.K339fs	p.Lys339fs	c.1016del	53.68%	P
P200	54	EC	MSH6	frameshift_variant	p.Y1066fs	p.Tyr1066fs	c.3195del	48.85%	LP
P204	54	EC	MSH6	frameshift_variant	p.S1257fs	p.Ser1257fs	c.3769del	50.56%	LP
P213	54	EC	FANCA	splice_acceptor_variant	c.1777-1G>C		c.1777-1G>C	39.06%	P
P216	58	EC	FANCL	splice_donor_variant	c.216+1G>T		c.216+1G>T	45.70%	LP
P221	56	EC	ATM	stop_gained	p.R23*	p.Arg23*	c.67C>T	45.72%	P
P231		EC	MSH6	frameshift_variant	p.R1172fs	p.Arg1172fs	c.3514dup	48.14%	P
P236	52	EC	RAD51C	stop_gained	p.R193*	p.Arg193*	c.577C>T	44.85%	P
P242	58	EC	BRCA1	stop_gained	p.E732*	p.Glu732*	c.2194G>T	50.60%	P
P249	48	EC	MSH2	frameshift_variant	p.L556fs	p.Leu556fs	c.1667del	44.37%	P

© 2023 Wen H et al. *JAMA Network Open.*

P253	60	EC	RAD51D	frameshift_variant	p.K91fs	p.Lys91fs	c.270_271dup	44.89%	P
P36	47	EC	MSH6	frameshift_variant	p.S285fs	p.Ser285fs	c.855_858del	51.08%	LP
P38	31	EC	MSH2	frameshift_variant	p.N566fs	p.Asn566fs	c.1697dup	50.81%	LP
P44	50	EC	NTHL1	frameshift_variant	p.S234fs	p.Ser234fs	c.699_700del	42.58%	LP
P45		EC	MLH1	splice_donor_variant	c.588dup		c.588dup	40.24%	LP
P46	48	EC	MSH2	frameshift_variant	p.N486fs	p.Asn486fs	c.1457_1460del	43.87%	P
P51	41	EC	PALB2	stop_gained	p.W1038*	p.Trp1038*	c.3113G>A	45.75%	P
P54	70	EC	MUTYH	stop_gained	p.R19*	p.Arg19*	c.55C>T	46.97%	P
P55	33	EC	PALB2	frameshift_variant	p.I1037fs	p.Ile1037fs	c.3108_3112del	67.92%	LP
P58	69	EC	BRCA1	frameshift_variant	p.L1306fs	p.Leu1306fs	c.3917dup	45.91%	LP
P59	51	EC	CHEK2	splice_donor_variant	c.444+1del		c.444+1del	46.77%	LP
P65	36	EC	MSH2	stop_gained	p.Q314*	p.Gln314*	c.940C>T	52.71%	P
P66	75	EC	BRCA2	stop_gained	p.G2274*	p.Gly2274*	c.6820G>T	59.31%	P
P68	57	EC	FANCI	splice_acceptor_variant	c.1113-1G>A		c.1113-1G>A	52.98%	LP
P8	42	EC	MSH2	splice_region_variant	c.942+3A>T		c.942+3A>T	45.89%	P
			EGFR	missense_variant	p.G863D	p.Gly863Asp	c.2588G>A	48.75%	LP
P82	59	EC	BRCA1	frameshift_variant	p.I1824fs	p.Ile1824fs	c.5470_5477del	47.04%	P
P85	59	EC	RAD51C	splice_acceptor_variant	c.706-2A>C		c.706-2A>C	46.12%	P
P87	37	EC	MSH2	frameshift_variant	p.T320fs	p.Thr320fs	c.958dup	47.99%	P
P90	43	EC	TP53	missense_variant	p.R158H	p.Arg158His	c.473G>A	11.61%	LP
P180	63	CC	BRCA2	stop_gained	p.R2318*	p.Arg2318*	c.6952C>T	48.99%	P
P151	61	CC	BRCA1	stop_gained	p.W1815*	p.Trp1815*	c.5445G>A	83.21%	P
P158	54	CC	BRCA1	missense_variant	p.L1786P	p.Leu1786Pro	c.5357T>C	50.94%	LP
P39	29	CC	BRCA2	frameshift_variant	p.A938fs	p.Ala938fs	c.2808_2811del	43.64%	P
P246	61	CC	RET	missense_variant	p.V804M	p.Val804Met	c.2410G>A	47.30%	P
P47	46	CC	FANCA	frameshift_variant	p.Q1307fs	p.Gln1307fs	c.3918dup	48.49%	LP
P32	36	CC	RAD54L	stop_gained	p.R96*	p.Arg96*	c.286C>T	40.28%	LP
P147	36	CC	RAD51B	frameshift_variant	p.N257fs	p.Asn257fs	c.770dup	49.71%	LP
P80	28	CC	BARD1	stop_gained	p.Y446*	p.Tyr446*	c.1338C>A	71.98%	LP
P6	50	CC	RAD51B	stop_gained	p.R8*	p.Arg8*	c.22C>T	40.06%	LP
P74	49	CC	RAD54L	frameshift_variant	p.D183fs	p.Asp183fs	c.537_547dup	37.95%	LP
P22	62	CC	BRCA2	frameshift_variant	p.M815fs	p.Met815fs	c.2442del	54.80%	P

© 2023 Wen H et al. *JAMA Network Open.*

P110	49	CC	MUTYH	missense_variant	p.R245H	p.Arg245His	c.734G>A	35.34%	LP
P27	41	CC	FANCA	splice_region_variant	c.709+5G>A		c.709+5G>A	48.09%	P
P227	65	CC	BRIP1	frameshift_variant	p.T997fs	p.Thr997fs	c.2990_2993del	43.70%	LP
P62	35	CC	BRCA2	stop_gained	p.S2670*	p.Ser2670*	c.8009C>A	74.80%	P
P202	45	CC	ATM	stop_gained	p.Q161*	p.Gln161*	c.481C>T	47.48%	LP
P75	34	CC	BRCA1	frameshift_variant	p.N1132fs	p.Asn1132fs	c.3395dup	47.44%	LP
P155	41	CC	FANCA	frameshift_variant	p.Q343fs	p.Gln343fs	c.1017_1027dup	41.94%	LP
P157	40	CC	FANCA	splice_acceptor_variant	c.3627-1G>A		c.3627-1G>A	46.53%	LP
P122	48	CC	RAD51C	large_genomic_rearrangement	exon6-9del		exon6-9del	1	LP
P252	42	CC	MSH2	large_genomic_rearrangement	exon6del		c.942+376_1076+222del	56.05%	LP
P205	57	CC	BRCA1	splice_region_variant			c.301+6T>C	13.66%	LP

Abbreviation: NA: not available; EC: endometrial cancer; OC: ovarian cancer; AF: allele frequency; P: pathogenic; LP: likely pathogenic

© 2023 Wen H et al. *JAMA Network Open.*

eTable 3. Distribution of Germline Pathogenic/Likely Pathogenic Variants Detected in the Cohort According to Genes and the Related Signaling Pathways

Pathway	Gene	Total	Ovarian	Endometrial	Cervical
Homologous recombination repair	BRCA1	118(44.5%)	110(55%)	4(9.5%)	4(17.4%)
	BRCA2	42(15.8%)	36(18%)	2(4.8%)	4(17.4%)
	RAD51D	15(5.7%)	14(7%)	1(2.4%)	0(0%)
	RAD51C	10(3.8%)	7(3.5%)	2(4.8%)	1(4.3%)
	FANCA	8(3%)	3(1.5%)	1(2.4%)	4(17.4%)
	BRIP1	6(2.3%)	5(2.5%)	0(0%)	1(4.3%)
	FANCI	5(1.9%)	2(1%)	3(7.1%)	0(0%)
	ATM	4(1.5%)	2(1%)	1(2.4%)	1(4.3%)
	PALB2	4(1.5%)	2(1%)	2(4.8%)	0(0%)
	RAD54L	4(1.5%)	0(0%)	2(4.8%)	2(8.7%)
	BARD1	2(0.8%)	1(0.5%)	0(0%)	1(4.3%)
	CHEK2	2(0.8%)	1(0.5%)	1(2.4%)	0(0%)
	RAD51B	2(0.8%)	0(0%)	0(0%)	2(8.7%)
	FANCL	1(0.4%)	0(0%)	1(2.4%)	0(0%)
Mismatch Repair	MSH2	14(5.3%)	4(2%)	9(21.4%)	1(4.3%)
	MSH6	7(2.6%)	1(0.5%)	6(14.3%)	0(0%)
	MLH1	4(1.5%)	2(1%)	2(4.8%)	0(0%)
	PMS2	1(0.4%)	1(0.5%)	0(0%)	0(0%)
Others	MUTYH	6(2.3%)	3(1.5%)	2(4.8%)	1(4.3%)
	MSH3	2(0.8%)	2(1%)	0(0%)	0(0%)
	NBN	1(0.4%)	1(0.5%)	0(0%)	0(0%)
	NTHL1	1(0.4%)	0(0%)	1(2.4%)	0(0%)
	RET	2(0.8%)	1(0.5%)	0(0%)	1(4.3%)
	EGFR	1(0.4%)	0(0%)	1(2.4%)	0(0%)
	SDHA	2(0.8%)	2(1%)	0(0%)	0(0%)
	TP53	1(0.4%)	0(0%)	1(2.4%)	0(0%)

© 2023 Wen H et al. *JAMA Network Open.*

eTable 4. Distribution of Germline Pathogenic/Likely Pathogenic Variants Detected from the Cohort According to Variant Type

Variant type	Total # of variants per variant type	Cancer type		
		Ovarian	Endometrial	Cervical
Total of mutation	265	200	42	23
Frameshift	129(48.7%)	105(52.5%)	16(38.1%)	8(34.8%)
Intronic variants	1(0.4%)	1(0.5%)	0(0%)	0(0%)
Large genomic rearrangement	7(2.6%)	5(2.5%)	0(0%)	2(8.7%)
Missense	24(9.1%)	17(8.5%)	4(9.5%)	3(13%)
Nonsense	72(27.2%)	52(26%)	13(31%)	7(30.4%)
Splice Site	30(11.3%)	18(9.0%)	9(21.4%)	3(13%)
Start loss	2(0.8%)	2(1%)	0(0%)	0(0%)

© 2023 Wen H et al. *JAMA Network Open.*
