# Supplementary material for: Prevalence and Landscape of Pathogenic or Likely Pathogenic Germline Variants and Their Association With Somatic Phenotype in Unselected Chinese Patients With Gynecologic Cancers
Source: JAMA Netw Open. 2023 Jul 31;6(7):e2326437. doi: 10.1001/jamanetworkopen.2023.26437 (PMC10391307; doi:10.1001/jamanetworkopen.2023.26437)
Supplement: Supplement 2. — Data Sharing Statement [file jamanetwopen-e2326437-s002.pdf]

Data Sharing Statement

Wen. Prevalence and Landscape of Pathogenic or Likely Pathogenic Germline Variants and Their Association With Somatic Phenotype in Unselected Chinese Patients With Gynecologic Cancers. *JAMA Netw Open*. Published July 31, 2023.
doi:10.1001/jamanetworkopen.2023.26437

Data
Data available: No
